# Effects of protein, muscle rehabilitation and trunk neuromuscular electrostimulation in patients with prolonged mechanical ventilation: a prospective, randomized controlled pilot study

**DOI:** 10.1080/07853890.2026.2728179

**Published:** 2026-09-22

**Authors:** Chun-Yu Lin, Po-Jui Chang, Meng-Jer Hsieh, Yu-Lun Lo, Shih-Wei Lin, Shu-Min Lin, Chen-Yiu Hung, Chun-Yu Lo, Ting-Yu Lin, Allen Chung-Cheng Huang, Ko-Wei Chang, Tsai-Yu Wang, Shih-Wei Huang, Jou-Yun Chang, Yu-Feng Chou, Chung-Chi Huang, Horng-Chyuan Lin

**Affiliations:** a Department of Thoracic Medicine, Kaohsiung Chang Gung Memorial Hospital, Kaohsiung, Taiwan; b College of Medicine, Chang Gung University, Taoyuan, Taiwan; c Department of Thoracic Medicine, Chang Gung Memorial Hospital at Linkou, Taoyuan, Taiwan; d Department of Nutrition, Chang Gung Memorial Hospital at Linkou, Taoyuan, Taiwan; e Department of Respiratory Therapy, Chang Gung Memorial Hospital at Linkou, Taiwan Taoyuan

**Keywords:** Protein supplementation, core muscle rehabilitation, neuromuscular electrostimulation, prolonged mechanical ventilation, weaning

## Abstract

**Background:**

Attenuating diaphragmatic dysfunction is important in patients with prolonged mechanical ventilation (PMV). The clinical impact of protein supplementation, core muscle rehabilitation and neuromuscular electrostimulation (NMES) in patients with PMV remains unknown.

**Study Design and Methods:**

We conducted a single center, prospective, randomized controlled pilot study for critically ill patients with PMV . All enrolled patients were randomly assigned into four groups: (1) usual care (UC) + high-protein diet (HP); (2) UC + HP + core muscle rehabilitation; (3) UC + HP + core muscle rehabilitation + NMES; and (4) UC. The interventions were maintained for 21 days. Primary outcomes were successful weaning from the ventilator and the duration of mechanical ventilation. Secondary outcomes were length of hospital stay and in-hospital mortality.

**Results:**

A total of 100 patients were enrolled, with 10 in the UC group and 30 each in the other three groups. Nine patients discontinued the trial early. The mean compliance rates of core muscle rehabilitation and NMES were nearly 90%. The patients in group 3 had the highest weaning rate followed by group 2 and group 1, and the difference was significant compared to group 4 (87%, 73%, 67% and 30%, *p* = 0.0071). The patients in group 3 also had the shortest duration of mechanical ventilation followed by group 1 and group 2, and the difference was significant compared to group 4 (41.1 ± 12.9, 56.5 ± 24.1, 57.1 ± 29.9 and 74.4 ± 27.1 days, *p* = 0.0006). The length of hospital stay and in-hospital mortality were comparable in the four groups.

**Conclusion:**

Combining high-protein supplementation, core rehabilitation, and NMES is feasible and may facilitate ventilator weaning in PMV patients. Larger multicenter randomized trials are warranted.

**Clinical Trial Registration Number:**

This trial was registered with ClinicalTrials.gov (NCT 05932134, Sep 2023) and is closed to new participants.

## Introduction

Critical illness is characterized by marked catabolism and muscle wasting, which are associated with intensive care unit (ICU)-acquired weakness and can lead to prolonged mechanical ventilation (PMV) [1,2]. Attenuating weakness of the diaphragm, the major respiratory muscle, is important and associated with weaning success [1,2].

Nutritional supplementation is quite important for muscle strengthening [3,4]. Higher protein supplementation improved clinical outcomes by attenuating protein loss and supporting the patients’ recovery by increasing respiratory muscle strength in the late phase of acute illness [3,4]. However, the optimal timing and dose of protein supplementation have yet to be determined. Full feeding in the early phase of acute illness can lead to overfeeding and detrimental effects [4]. Energy intake at 70–80% of the measured resting energy expenditure was associated with a survival advantage [5]. Several large randomized controlled trials reported that higher protein delivery (1.5–2.0 g/kg/day) in the early phase of ICU stay did not improve clinical outcomes, and that it was even associated with increased mortality among patients with acute kidney injury (AKI) [3,6,7]. The timing of protein administration may be relevant in determining outcomes, especially in the anabolic phase [3,4]. Koekkoek et al. reported that protein intake more than 1.2 g/kg/day after day 5 was associated with the lowest 6-month mortality rate [8]. Further larger observational studies also found that moderate protein intake (0.8–1.2 g/kg/day) was associated with the most significant survival benefit in the late acute phase of critical illness [9–11].

In addition to nutritional support, physical activity may have synergistically beneficial effects in reducing morbidity [4]. Higher protein targets combined with exercise have been shown to potentially benefit physical activity and survival in patients with PMV [12,13], and attenuating diaphragm weakness [14]. Inspiratory muscle training has been demonstrated to improve diaphragm function by increasing the maximal inspiratory pressure, consequently improving weaning outcomes [15–18]. Although inspiratory muscle training is usually effective in patients who are cooperative, most patients with PMV have altered cognitive status [18]. Dres et al. used a multielectrode stimulating central venous catheter to induce temporary bilateral phrenic stimulation to strengthening diaphragm, but resulted in no weaning benefits in difficult-to-wean patients [19]. The diaphragm is not only a respiratory muscle but also plays a crucial role in postural stability [20]. Core muscle and trunk stabilization may potentially progressively restore weakness of the diaphragm muscle [20,21]. Thus, we assumed that core muscle training may strengthen diaphragm muscles, and consequently improve weaning outcome.

Neuromuscular electrical stimulation (NMES) is an effective management strategy for deficient muscles that does not require patient cooperation, providing functional advantages for critically ill patients [22]. NMES applied to the abdominal and back muscles has been demonstrated to improve trunk balance [23,24]. Furthermore, abdominal functional electrical stimulation (FES) may augment respiratory function, assist in ventilator weaning, and facilitate tracheostomy decannulation by activating key expiratory muscles [25–27]. While NMES can reduce general muscle weakness in critical illness [28], its specific synergistic effect with core rehabilitation in PMV patients remains unexplored.

Here, we conducted a randomized controlled pilot study and aimed to investigate the weaning benefits of combining high-protein supplementation, core muscle rehabilitation and NMES in patients with PMV.

## Material and methods

This randomized controlled trial was conducted at a 24-bed respiratory care center (RCC) at Linkou Chang Gung Memorial Hospital in Taiwan from September 18, 2023 to September 18, 2024. RCC is a special unit that accepts patients requiring mechanical ventilation ≥21 days under stable condition (hemodynamic stability, free of sedative agents) but could not have successful weaning from the ventilator in the ICU for 21 days. Patients in RCC receive medical treatment of active disease, causes of respiratory failure, limb rehabilitation, and ventilator weaning. There was a standardized weaning protocol in RCC, and the detailed steps of the protocol were described in previous publications [29]. The inclusion criteria were as follows: 1) age ≥ 20 years; 2) using mechanical ventilator for more than 21 days in ICU (including patients with tracheostomy or endotracheal tube); 3) stable clinical condition, without using inotropic agents, having overcome the early period of hemodynamic instability; 4) maximal inspiratory pressure < 30 mmHg; and 5) receiving enteral nutrition *via* a nasogastric tube. The exclusion criteria were patients with: 1) acute infection and sepsis (fever up to 38.5 °C); 2) severe neuromuscular disease; 3) uncontrolled epilepsy; 4) bony fractures or a history of deep vein thrombosis; 5) wounds over the abdomen; 6) congestive heart failure with ejection fraction < 40% or using a pacemaker; 7) body mass index > 35 kg/m^2^; 8) severe edema; 9) hepatic failure; 10) rapidly progressing malignancy; or 11) pregnancy. We obtained written informed consent from the patients or their legally acceptable surrogate. The study protocol was approved by independent medical ethics committees of Chang Gung Medical Foundation (approval no. 202300154A3). The study protocol was registered at ClinicalTrials.gov (NCT 05932134, Sep 18, 2023).

### Study protocol

According to the order of enrollment, the patients were randomly assigned into four groups: (1) usual care (UC) + high-protein diet (HP); (2) UC + HP + core muscle rehabilitation; (3) UC + HP + core muscle rehabilitation + NMES; (4) UC. A pre-specified 3:3:3:1 randomization scheme was adopted based on the pilot and exploratory nature of this study. Because high-protein supplementation was considered a fundamental component of medical nutrition therapy in our clinical setting, the trial was designed to primarily investigate the feasibility and potential incremental benefits of core rehabilitation and trunk NMES when combined with nutritional support. Therefore, a larger sample size (*n* = 30 per group) was allocated to the three active intervention groups to ensure adequate experience with protocol adherence and safety profiling, while a smaller cohort (*n* = 10) was maintained for the usual-care control group as an exploratory baseline. Ten patients were included in the UC group, with 30 each in the other three groups (Figure 1). The patients were randomized using a table of random numbers and sealed envelopes. After randomization, we initiated the interventions during the first three days and maintained them for 21 days. After 21 days intervention, if the patient was still supported by mechanical ventilator, we kept the standardized weaning protocol and care in RCC [29]. The UC group received nutritional supplementation according to the ESPEN guideline [4] and usual physical therapy (i.e. passive four limbs mobilization and stretching) as our RCC weaning protocol [29]. The total daily caloric intake of the HP group was based on the suggestion of a nutritionist, and the protein content was increased to 1.5 g/kg/day (according to ideal body weight). The specific formulas were chosen for their different energy and protein contents and according to the patients’ digestion. The protein content was not reduced in patients with renal failure. We recorded daily data of actual caloric and protein intake for 21 days or until discharge or death. Core muscle rehabilitation was performed with the patient sitting up (around 90°, slightly forward, in a core muscle activation position [30]) with or without aids, for 30 min, twice per day, 5 days per week, for 21 days. NMES was also applied for 30 min, twice per day, 5 days per week, for 21 days *via* surface rectangular electrodes (5 cm × 10 cm). The electrodes were placed on the back to activate the latissimus dorsi, and the abdominal wall to activate the transversus abdominis and internal and external oblique muscles, as previously described [27] (Figure 2). Electrical muscle stimulation was performed using a commercial stimulator (GEMORE, GM300E, Taipei, Taiwan) with biphasic waves at a stimulation frequency of 30 Hz and pulse width of 400 μs, cycling 2 s on and 4 s off. Electrical muscle stimulation intensity was gradually increased until a visible muscle contraction was observed (median 50 mA [range 30–60 mA]). In conscious patients, intensity was adjusted according to their tolerance.

**Figure 1.**
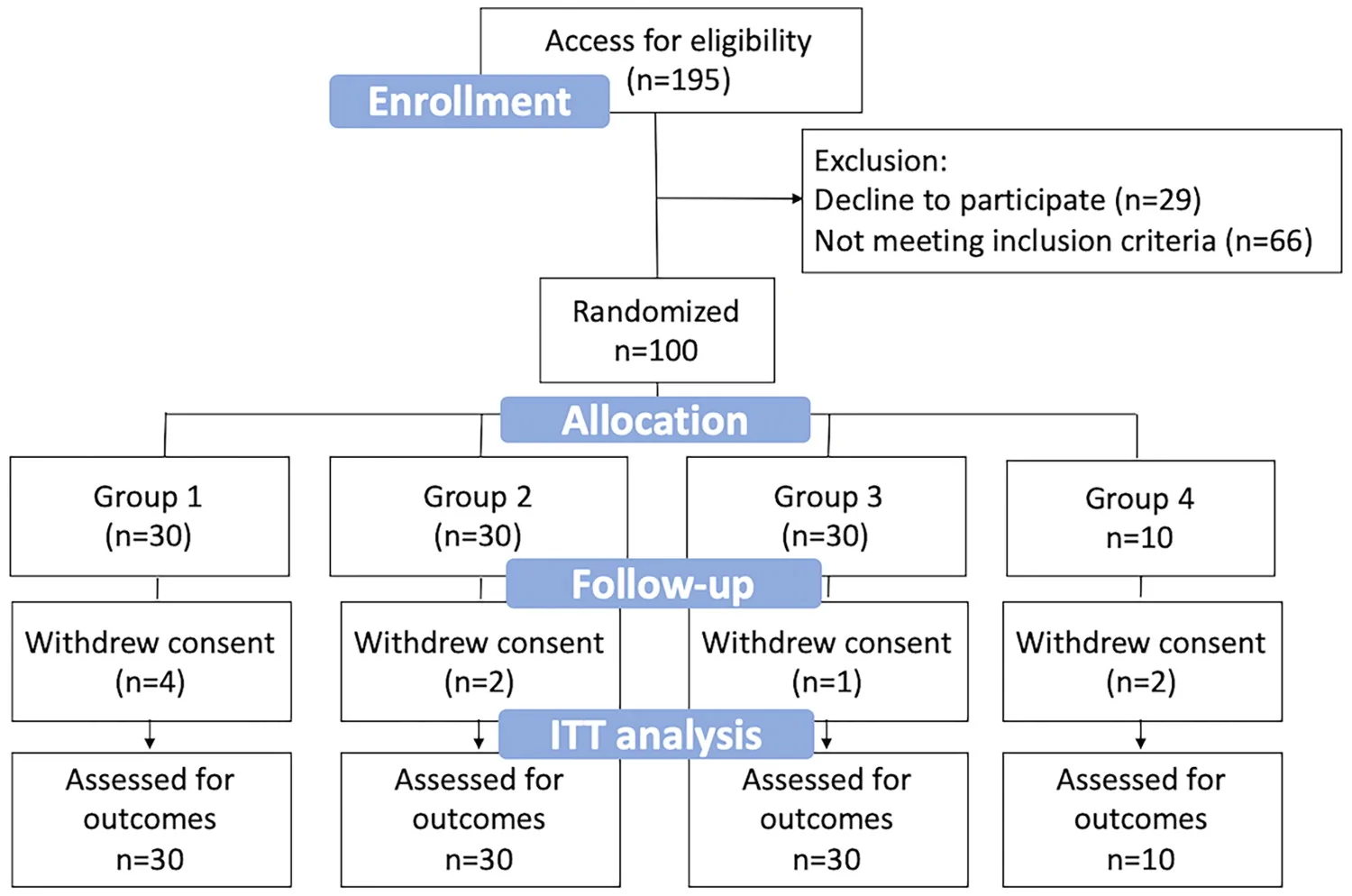
Flow chart of the study population.

**Figure 2.**
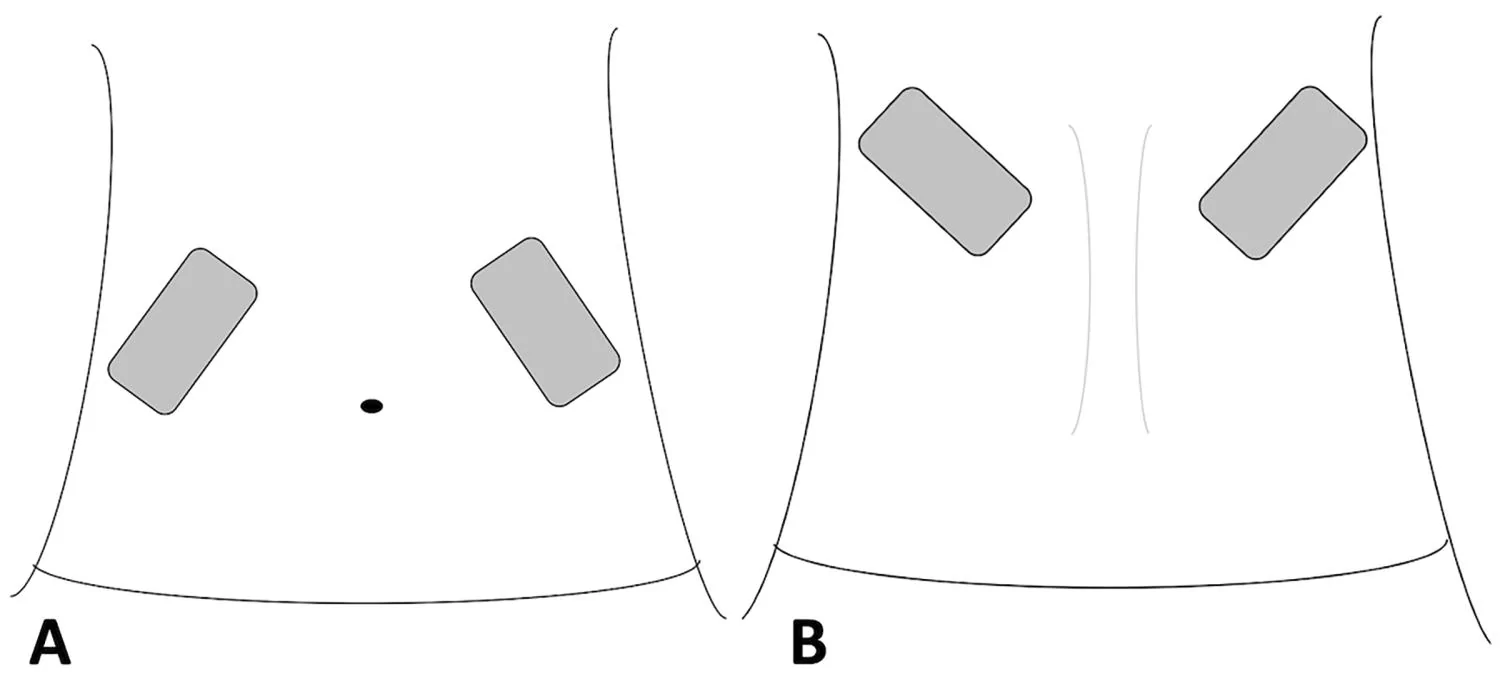
Schematic diagram of neuromuscular electrostimulation (NMES) electrode placement: (A) on the lateral abdominal wall, placed 2 cm below and parallel to the costal margin from the midline, angled diagonally downward in an inferolateral direction, towards the anterior superior iliac spine; (B) electrode placement on the latissimus dorsi.

### Outcome measures

Age, sex, smoking history, diagnosis, duration of mechanical ventilation, and acute physiology and chronic health evaluation (APACHE) II score were recorded. Pulmonary performance including tidal volume, maximal inspiratory pressure (PiMax), rapid shallow breathing index (RSBI), lung compliance, airway resistance, blood gas analysis, Barthel index, serum albumin, creatinine, and electrolytes was recorded before, during (twice per week) and after the intervention. Nitrogen balance was calculated using daily protein intake from enteral feeds and the measured urine urea nitrogen excretion from the collection of 24 h urine (24 hr UUN). The formula was: Nitrogen balance = (grams protein intake/6.25) - (24hrUUN + 4) [31].

The primary outcomes were successful weaning from the ventilator and the duration of mechanical ventilation after the intervention among the four groups. Successful weaning was defined as breathing without mechanical ventilatory support for 5 consecutive days [29]. The secondary outcomes were length of hospital stay and in-hospital mortality. Discharge and in-hospital mortality were followed up to 90 days after the intervention.

Adverse events were recorded in daily electronic record files and reported to the ethical review boards. Study-emergent adverse events were immediately reported to the principal investigators and medical ethical committees.

### Statistical analysis

An a priori power analysis based on an ANOVA framework estimated that 100 patients would provide 80% power to detect a moderate effect size (*f* = 0.25). However, as a pilot study focused primarily on feasibility and safety, a pragmatic 3:3:3:1 allocation was employed to maximize intervention exposure in the active groups. Consequently, efficacy outcomes, including weaning success, should be interpreted as preliminary. The primary analysis included all patients who underwent randomization, included in the analysis according to the intention-to-treat principle and irrespective of adherence to the protocol and continued trial participation (Figure 1). Categorical variables were expressed as count and percentage, and continuous variables were expressed as mean and standard deviation. Compliance was defined as the number of sessions completed as a percentage of all sessions that should have been completed during the study period. Changes in the volume parameters, pulmonary function parameters and disease severity were tested using the Kruskal–Wallis test between groups. When a significant overall difference was observed, post-hoc pairwise comparisons were performed using Dunn’s multiple comparisons test, and adjusted p-values were reported to control the family-wise error rate. Kaplan–Meier survival analysis was performed to assess weaning and survival outcomes. The Mann-Whitney test was used for comparing non-parametric data (including weaning rate, duration of ventilator use, length of ICU stay and length of hospitalization). The Mann-Whitney U test was reserved for pre-specified independent two-group comparisons where appropriate. Statistical significance was set at a p-value < 0.05. Statistical analyses were performed using GraphPad Prism version 8 (GraphPad Software, La Jolla, CA, USA) and SPSS version 26 (IBM Inc., Armonk, NY, USA).

## Results

A total of 195 patients were admitted to the RCC during the study period, of whom 66 did not meet the inclusion criteria and 29 declined to participate. We randomized the remaining 100 patients into the four study groups: Group 1, UC + HP (*n* = 30); Group 2, UC + HP + core muscle rehabilitation (*n* = 30); Group 3, UC + HP + core muscle rehabilitation + NMES (*n* = 30); and Group 4, UC (*n* = 10).

Among the 100 randomized patients, nine patients (9%) discontinued the trial early. The distribution of these withdrawals was as follows: 4 patients in Group 1, 2 in Group 2, 1 in Group 3, and 2 in Group 4. The specific reasons for discontinuation included family concerns regarding the high-protein diet’s potential effect on renal function (*n* = 4), uncooperative or violent behavior (*n* = 2), and irritable mood preventing rehabilitation (*n* = 3). We compared the baseline demographic and clinical severity parameters between the 91 protocol completers and the 9 non-completers, and found no significant differences (all *p* > 0.05), suggesting that the attrition did not introduce selection bias. Finally, 91 patients completed the study protocol. However, according to the intention-to-treat principle, all 100 randomized patients were included in the primary outcome analyses (Figure 1).

### Clinical characteristics

The demographic and clinical data were comparable between the four groups (Table 1). There were no significant differences in age, sex, APACHE II score, sequential organ failure assessment (SOFA) score, mNUTRIC score, body mass index, ICU length of stay before transferred to RCC, main reason for ICU admission, daily protein intake, serum creatinine and albumin levels between the four groups. After the interventions, only the patients in group 3 had improved daily caloric intake (from 24.2 ± 5.4 kcal/kg/day to 27.3 ± 6.3 kcal/kg/day, *p* = 0.002, Figure 3A). The actual daily protein intake significantly improved after the interventions in group 1, group 2 and group 3 compared to group 4 (from 1.2 ± 0.4 g/kg/day to 1.4 ± 0.3 g/kg/day, *p* = 0.0025; from 1.3 ± 0.4 g/kg/day to 1.5 ± 0.3 g/kg/day, *p* = 0.0008; from 1.3 ± 0.4 g/kg/day to 1.4 ± 0.4 g/kg/day, *p* = 0.041; versus from 1.3 ± 0.8 g/kg/day to 1.2 ± 0.3 g/kg/day, *p* = 0.487; respectively, Figure 3B). Both nitrogen balance (Figure 3C) and serum creatinine levels (Table 1) were similar in the four groups before and after the interventions.

**Figure 3.**
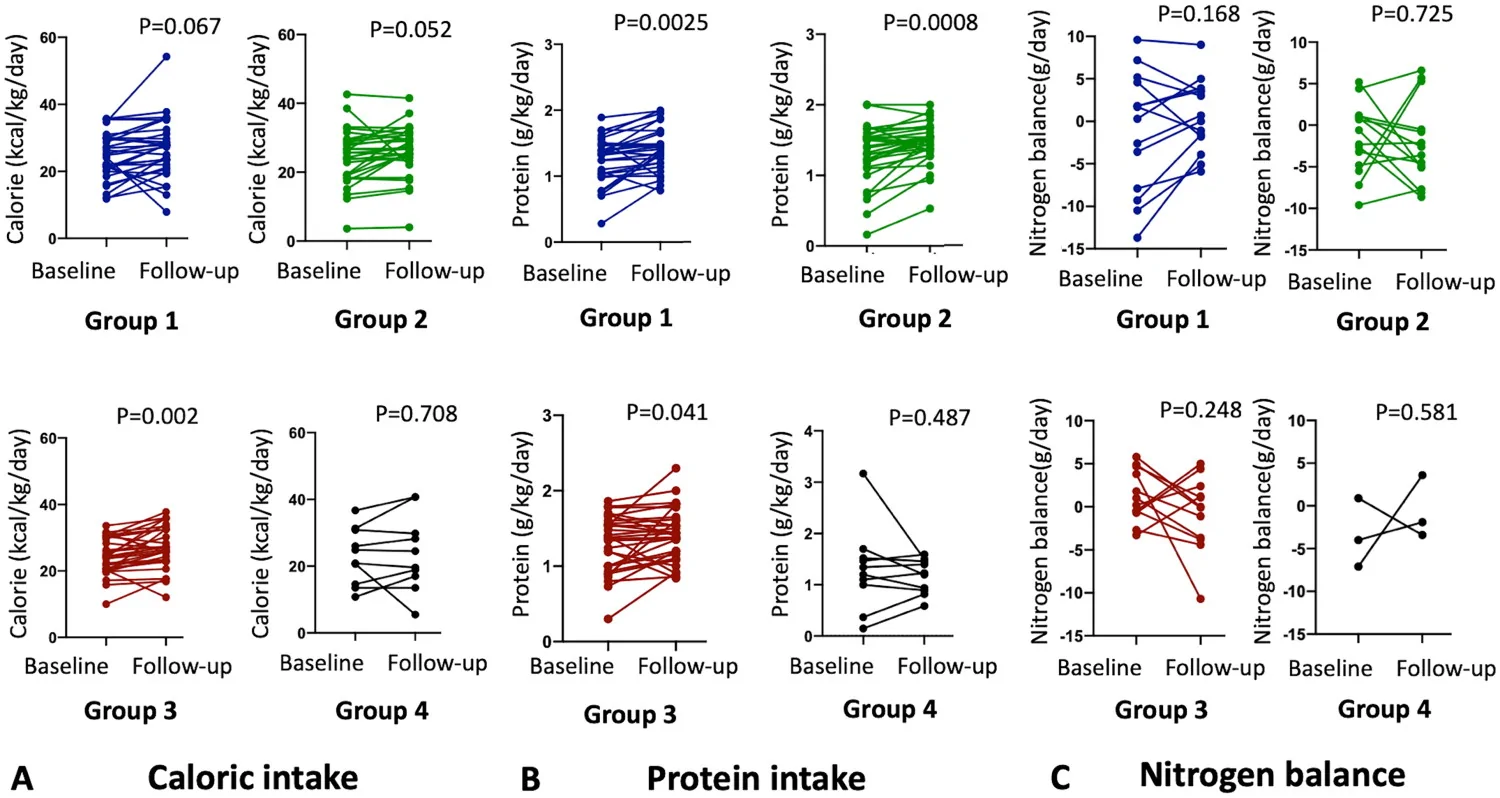
Comparison of nutritional parameters of the study population before and after the interventions: (A) daily caloric intake, (B) daily protein intake, (C) nitrogen balance.

**Table 1.** Clinical characteristics of the patients with PMV.

Characteristics	Group 1 *n* = 30	Group 2 *n* = 30	Group 3 *n* = 30	Group 4 *n* = 10	*p* value
Age, years	69.3 ± 11.9	67.2 ± 12.1	71.5 ± 9.1	72.8 ± 10.9	0.552
Male	16, 53%	16, 53%	19, 63.3%	8, 80%	0.415
APACHE II score	22.1 ± 8.7	21.1 ± 9.7	20.2 ± 7.9	23.3 ± 6.1	0.694
SOFA score	8.5 ± 2.3	8.3 ± 2.4	8.7 ± 2.2	7.9 ± 2.8	0.455
mNUTRIC score	5.1 ± 1.4	4.8 ± 1.8	5.4 ± 1.8	5.3 ± 1.3	0.614
BMI (kg/m^2^)	24.0 ± 5.8	25.5 ± 7.5	23.0 ± 4.6	23.2 ± 4.3	0.601
ICU length of stay, days (before transferred to RCC)	25.7 ± 7.9	26.0 ± 8.3	23.7 ± 7.5	31.3 ± 12.8	0.116
Main reason for ICU admission					0.567
Respiratory	19 (63%)	15 (50%)	21 (70%)	6 (60%)	
Infection	4 (13%)	4 (13%)	3 (10%)	1 (10%)	
Cardiac	5 (17%)	2 (7%)	5 (17%)	2 (20%)	
Neurological	7	12	8	0 (0)	
Others	4 (13%)	4 (13%)	1 (3%)	2 (20%)	
Baseline daily protein, g/kg/day	1.2 ± 0.4	1.3 ± 0.4	1.3 ± 0.4	1.3 ± 0.8	0.132
Follow-up daily protein, g/kg/day	1.4 ± 0.3	1.5 ± 0.3	1.4 ± 0.4	1.2 ± 0.3	0.127
Baseline daily calories, kcal/kg/day	24.5 ± 6.9	24.3 ± 8.0	24.2 ± 5.4	23.0 ± 8.5	0.978
Follow-up daily calories, kcal/kg/day	26.7 ± 8.9	26.5 ± 7.2	27.3 ± 6.3	23.8 ± 11.3	0.744
Baseline nitrogen balance	−0.7 ± 6.5	−1.7 ± 3.9	0.9 ± 3.9	−1.0 ± 5.3	0.467
Follow-up nitrogen balance	0.7 ± 4.2	−2.2 ± 5.0	−0.8 ± 4.3	−0.6 ± 3.7	0.453
Baseline creatinine, mg/dL	1.6 ± 1.7	1.1 ± 1.4	1.6 ± 1.6	1.3 ± 1.4	0.168
Follow-up creatinine, mg/dL	1.6 ± 1.6	1.3 ± 1.2	1.5 ± 0.9	1.3 ± 1.2	0.621
Baseline albumin, g/dL	3.0 ± 0.5	3.1 ± 0.4	2.9 ± 0.5	2.7 ± 0.4	0.144
Follow-up albumin, g/dL	3.1 ± 0.4	3.3 ± 0.3	2.8 ± 0.5	2.5 ± 0.4	0.132
Baseline PiMax, cm H_2_O	−35.3 ± 13.8	−31.8 ± 12.5	−31.3 ± 10.5	−28.6 ± 13.9	0.358
Follow-up PiMax, cm H_2_O	−36.8 ± 12.3	−35.9 ± 11.2	−36.1 ± 14.8	−29.5 ± 7.5	0.462
Baseline RSBI	70.1 ± 30.8	90.1 ± 61.6	78.4 ± 43.6	120.6 ± 57.3	0.068
Follow-up RSBI	90.6 ± 76.5	72.8 ± 34.2	71.4 ± 37.3	70.2 ± 16.6	0.962
Baseline Barthel index	2.8 ± 6.9	0.8 ± 3.7	3.5 ± 11.9	2.5 ± 7.9	0.671
Follow-up Barthel index	5.2 ± 10.3	1.8 ± 6.1	6.2 ± 18.1	2.5 ± 7.9	0.660

*Notes:* Data are mean ± SD, or n (%), unless otherwise specified.

Groups: group 1: UC + HP; group 2: UC + HP + core muscle rehabilitation; group 3: UC + HP + core muscle rehabilitation + NMES; group 4: UC.

Abbreviations: PMV, prolonged mechanical ventilation; APACHE, acute physiology and chronic health evaluation; SOFA, sequential organ failure assessment; BMI, body mass index; ICU, intensive care unit; RCC, respiratory center; RSBI, rapid shallow breathing index; UC, usual care; HP, high-protein diet; NMES, neuromuscular electrostimulation.

The mean compliance with the core muscle training and NMES sessions were 89.2% and 88.9% in group 2 and group 3, respectively. The mean duration of the intervention was 15.3 ± 5.6 days in group 2 and 12.0 ± 5.9 days in group 3. No adverse events occurred during the study period. Seventeen of the patients died, however none of them were related to the intervention and none occurred during the intervention period.

### Outcomes

Regarding the primary outcomes, the patients in group 3 had the highest weaning rate (26 of 30, 87%), followed by group 2 (22 of 30, 73%) and group 1 (20 of 30, 67%), and the difference was significant compared to group 4 (3 of 10, 30%, *p* = 0.0071, Table 2). The patients in group 3 also had the shortest duration of mechanical ventilation (41.1 ± 12.9 days), followed by group 1 (56.5 ± 24.1 days) and group 2 (57.1 ± 29.9 days), and the difference was significant compared to group 4 (74.4 ± 27.1 days, *p* = 0.0006, Table 2, Figure 5). Post-hoc pairwise comparisons using Dunn’s test revealed that patients in group 3 had a significantly shorter duration of mechanical ventilation compared to group 4 (adjusted *p* = 0.0006). Patients in group 2 had a comparable duration of mechanical ventilation to group 1 patients (adjusted *p* = 0.481) and showed a trend toward a shorter duration when compared to group 4 (adjusted *p* = 0.0947). Although it did not reach statistical significance, patients in group 1 also demonstrated a trend toward a shorter duration of mechanical ventilation compared to group 4 (adjusted *p* = 0.0962). Regarding the secondary outcomes, the length of hospital stay, length of ICU stay and in-hospital mortality were similar in the four groups (Table 2). The baseline RSBI of group 4 was higher than in the other groups, but the difference did not reach significance (Table 1, Figure 4). The PiMax, RSBI and Barthel index before and after the interventions were comparable between the four groups (Table 1, Figure 4).

**Figure 4.**
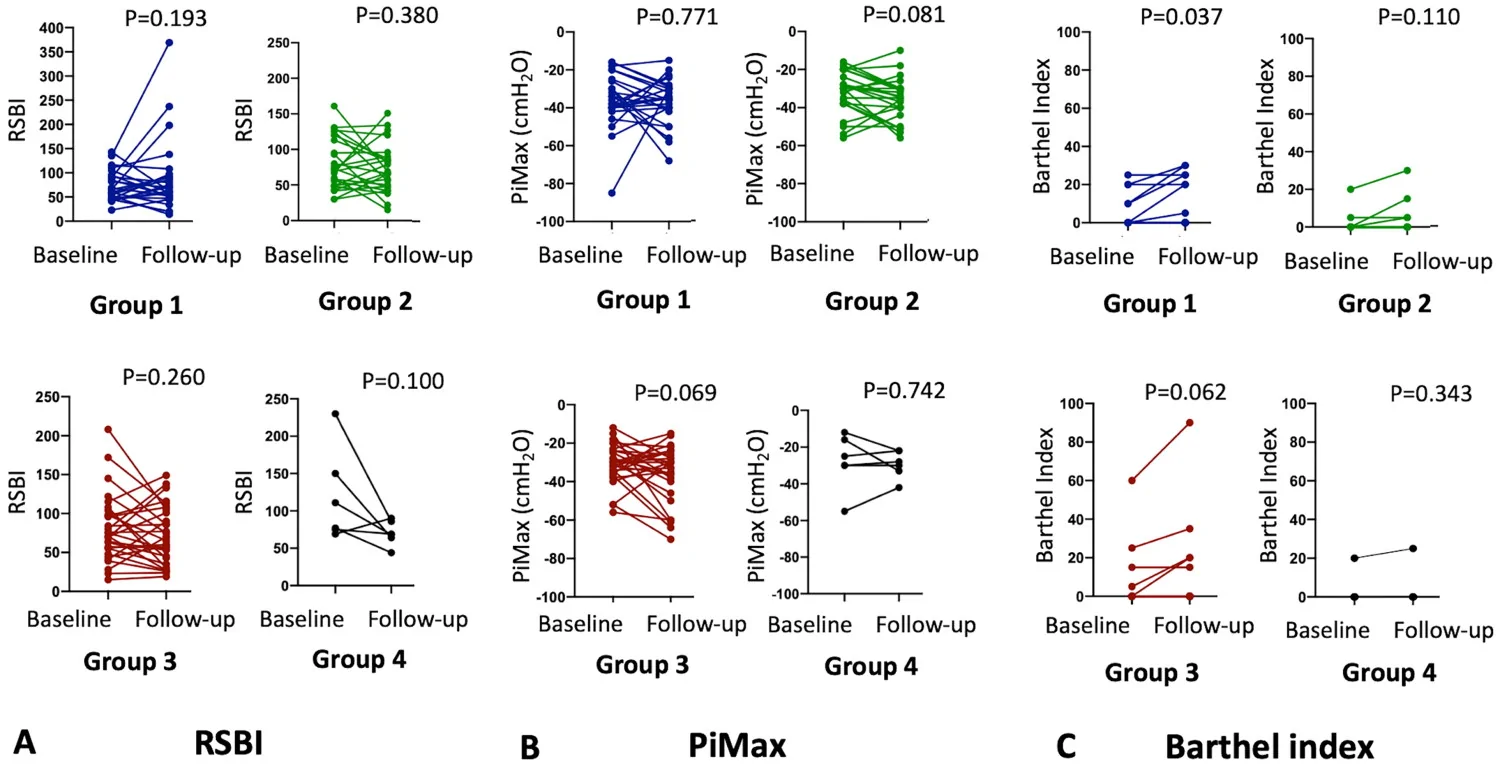
Comparison of weaning parameters and performance status of the study population before and after the interventions: (A) rapid shallow breathing index, (B) maximum inspiratory pressure (PiMax), (C) Barthel index.

**Figure 5.**
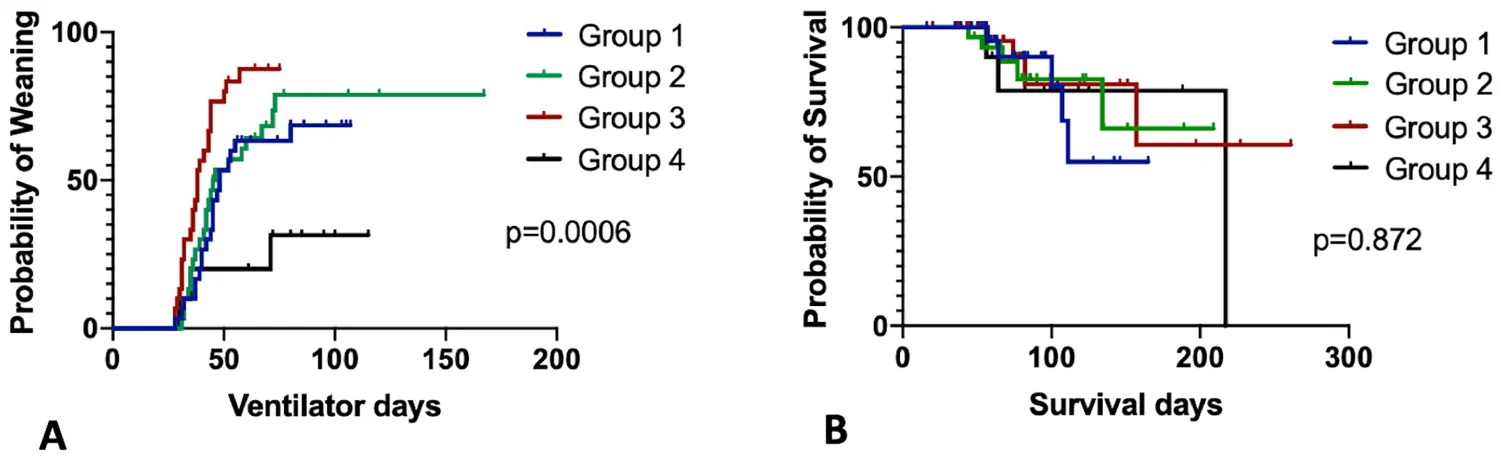
Outcomes in the four groups: (A) duration of mechanical ventilation, (B) overall survival curves. Notes: *p*-values for multiple group comparisons were calculated using the Kruskal-Wallis test, followed by Dunn’s post-hoc test for adjusted p-values.

**Table 2.** Outcomes of the patients with PMV.

	Group 1 *n* = 30	Group 2 *n* = 30	Group 3 *n* = 30	Group 4 *n* = 10	*p* value
Hospital length of stay, days	81.5 ± 34.9	89.6 ± 40.1	92.1 ± 57.5	110.4 ± 54.2	0.872
Length of mechanical ventilation, days	56.5 ± 24.1	57.1 ± 29.9	41.1 ± 12.9	74.4 ± 27.1	0.0006
Weaning success	20 (67%)	22 (73%)	26 (87%)	3 (30%)	0.0071
In-hospital mortality	5 (17%)	5 (17%)	4 (13%)	3 (30%)	0.685

*Notes:* Data are mean ± SD, or n (%), unless otherwise specified.

Groups: group 1: UC + HP; group 2: UC + HP + core muscle rehabilitation; group 3: UC + HP + core muscle rehabilitation + NMES; group 4: UC.

Abbreviations: PMV, prolonged mechanical ventilation; ICU, intensive care unit; UC, usual care; HP, high-protein diet; NMES, neuromuscular electrostimulation.

## Discussion

To the best of our knowledge, this is the first study to investigate the clinical effects of high protein supplementation coupled with core muscle rehabilitation and NMES in patients with PMV (duration of mechanical ventilation ≥ 21 days). In this prospective, randomized controlled pilot study, we found a significant improvement in weaning success and a significant reduction in the duration of mechanical ventilation in the patients who received high-protein supplementation, core muscle rehabilitation and/or with NMES. Compliance with the intervention sessions was nearly 90% in the core muscle rehabilitation and NMES groups, and no adverse effects were recorded. These findings demonstrate the feasibility and safety of applying these interventions in patients with PMV and relatively stable status.

Ventilator-induced diaphragmatic dysfunction along with ICU-related neuromuscular dysfunction can lead to weaning difficulty [32,33]. Protein supplementation in critically ill patients has been shown to restore muscle strength in the late phase of ICU stay [3,4]. Nevertheless, full feeding in the early phase of acute illness can lead to overfeeding and deleterious effects [4,5]. Recently, the emerging concept of ‘nutritional stewardship’ and the optimization of medical nutrition therapy have been highlighted as critical strategies in the intensive care setting. Optimized nutrition is essential not only for meeting metabolic demands but also for preserving muscle mass and facilitating timely ventilator liberation in critically ill patients [34–36]. Bels et al. and Heyland et al. reported negative findings of high-protein supplementation in the early phase of ICU stay in large, international, multicenter studies [6,7]. Moreover, high protein intake has been reported to potentially increase the risk of mortality in patients with AKI [3,7]. Koekkoek et al. reported that thoroughly low protein intake and high protein intake during the first 3 to 5 days of ICU stay were associated with increased mortality [8]. In comparison, early moderate daily protein supplementation has been associated with improved clinical outcomes [8,10]. The ESPEN guidelines also recommend avoiding early full nutritional feeding, and that it should be applied progressively in the late acute phase (3^rd^ to 7^th^ days) [4]. In our previous study, we demonstrated that higher protein supplementation may be beneficial in weaning patients with PMV (≥ 21 days), and that it did not negatively impact renal function [29]. The timing of protein administration has been suggested to affect the outcomes in patients with PMV [3,4]. In this prospective, randomized controlled pilot study, we selected patients who were in a relatively stable condition and had received mechanical ventilation for more than 21 days. We found that administering high-protein supplementation (actual feeding with 1.4–1.5 g/kg per day) resulted in a trend towards a lower duration of mechanical ventilation compared to those who received usual nutritional care. These findings suggest that the beneficial effects of protein supplementation may be restricted to patients who are in a stable anabolic state.

Physical activity may have synergistically beneficial effects with nutritional support [3,4]. Azevedo et al. conducted a prospective randomized controlled trial to investigate the clinical effects of high protein intake and early exercise (HPE) [12]. The daily protein intake in the HPE group was 1.48 g/kg, and they received two daily sessions of cycle ergometry exercise. The daily protein intake was 1.19 g/kg in the control group, who also received routine physiotherapy, and the results showed better survival in the HPE group compared to the control group [12]. On the other hand, Kagan et al. found non-significant benefits in outcomes between patients who received cycle ergometry combined with protein-enriched enteral nutrition and those who received conventional physiotherapy with standard nutrition [34,35]. However, they enrolled patients who had been mechanically ventilated for 48 h, so they may not have been in a stable condition [34,35]. Moreover, the effects of cycle ergometry in strengthening the diaphragm may be limited. The diaphragm is not only the strongest respiratory muscle but it also plays an important role in maintaining postural stability, and its activation stabilizes the trunk [20]. Dülger et al. reported increased diaphragm muscle thickness after trunk stabilization exercises [21]. In addition, core stabilization training has been suggested to potentially progressively restore weakness of the diaphragm muscle and improve weaning success [20]. Theoretically, abdominal and back muscles would be activated in the sitting position, including the transversus abdominis and internal and external oblique muscles, multifidus muscle and latissimus dorsi. Sitting straight up, increasing abdominal pressure and increasing work of breathing may ameliorate diaphragm dysfunction and improve weaning from mechanical ventilation [36,37]. In the current study we included patients who had been bedridden for a prolonged period, and the core muscle rehabilitation program involved sitting straight up (around 90°, slightly forward, in a core muscle activation position [30]) with or without aids, to passively or actively contract abdominal and back muscles. The patients in group 2 received a combination of high-protein supplementation and core muscle rehabilitation, and they had a significantly improved weaning rate and shorter length of mechanical ventilation. No adverse effects occurred during the intervention. Taken together, high protein supplementation combined with simple core muscle rehabilitation is a feasible and safe method that may be able to ameliorate diaphragm weakness and enhance the weaning process.

Many studies have demonstrated that NMES in critically ill patients may reduce limb muscle weakness, but its impact on reducing the mechanical ventilator duration, length of ICU stay or mortality remained inconclusive [28,38,39]. However, most previous NMES studies focused on leg or limb muscles and did not directly target respiratory muscles. Ko et al. reported that a combination of trunk NMES of abdominal and back muscles and core muscle strengthening had synergistic effects in trunk balance outcomes [23]. McCaughey et al. conducted a double-blinded, randomized, sham-controlled pilot study and recruited 20 critically ill mechanically ventilated patients to investigate the effects of FES in abdominal muscles, which could theoretically enhance respiratory function [25–27]. More than 90% of the patients finished the FES sessions, and although there was no difference in diaphragm thickness between the FES and control groups, the patients in the abdominal FES group had a shorter mechanical ventilation duration and ICU length of stay [27]. Nevertheless, NMES may have unsatisfactory effects in muscle response in patients presenting with higher APACHE II score and showed limited effects during acute stage [22]. In the current study, the enrolled patients were in subacute phase and under relative stable condition. The patients in group 3 received NMES of abdominal muscles, as mentioned by McCaughey et al. [27], and the latissimus dorsi muscle while they were sitting straight up (around 90°, slightly forward) with or without aids, contracting the trunk muscles. The compliance in group 3 was nearly 90%, without any reported complications. The patients received a high-protein diet, core muscle rehabilitation and NMES of trunk muscles, and they had the highest weaning rate (87%) and shortest duration of mechanical ventilation (41.1 ± 12.9 days). Our approach strongly aligns with the current paradigms of nutritional stewardship, which advocate for optimizing medical nutrition therapy to reduce the duration of mechanical ventilation [34,37,38]. By providing high-protein supplementation to all active intervention groups, we aimed to establish a necessary metabolic foundation to support muscle anabolism and maximize the functional benefits of core rehabilitation and NMES.

An important mechanistic observation in our study relates to the trajectory of the rapid shallow breathing index (RSBI) shown in Figure 4 and Table 1. Although the usual-care group (Group 4) presented with a higher, borderline-significant baseline RSBI, the follow-up RSBI values measured before weaning were highly comparable across all four groups without statistical difference (*p* = 0.962, Table 1, Figure 4).

However, despite achieving this favorable physiological parameter, their actual weaning success rate remained disproportionately low (30%) compared to the active intervention groups. This phenomenon strongly supports our core hypothesis: while optimizing respiratory parameters is necessary, it is often insufficient on its own for successful ventilator liberation in PMV patients. The functional capacity and muscle strength provided by core muscle rehabilitation and NMES appear to be indispensable components for translating physiological readiness into actual weaning success.

In summary, the results of this study showed that high-protein supplementation alone did not have a significant effect on the duration of mechanical ventilation. However, when combined with core muscle rehabilitation and/or NMES of core muscles, the weaning success rate and duration of mechanical ventilation significantly improved. Nutritional support may be the key to augment the effects of rehabilitation and NMES, in reducing the detrimental effects of critical illness. These interventions are minimally invasive, simple, feasible and safe for patients with PMV, and they may provide significant benefits in weaning patients from mechanical ventilation.

There are several limitations to this study. First, the trial was conducted at the RCC of a single center. In addition, the nutritional protocol and interventions were not blinded to the staff, which may have introduced bias. Second, we calculated caloric and protein intake using body weight according to the ESPEN guidelines rather than calorimetry to calculate energy expenditure. Consequently, nutritional support may not have been adequate for the patients. A third notable limitation is the absence of direct measurements regarding diaphragm structure or function. Because we did not perform diaphragmatic ultrasound, thickening fraction, excursion measurements, or electromyographic assessments, and given that PiMax did not significantly improve across groups, the exact physiological mechanisms linking our interventions to improved weaning outcomes remain speculative. Fourth, we enrolled a relatively small number of patients in each group, which may limit the validity of the results. Moreover, the study employed an unequal 3:3:3:1 allocation ratio, with only 10 patients in the usual-care control group. While this design allowed us to focus resources on exploring the feasibility and adherence of the novel combination therapies in the active groups, the small sample size of the control group substantially limits the statistical power for direct statistical comparisons with the control arm and may introduce selection bias. Due to the small sample size and the limited number of weaning events in the control group, this study was unable to perform robust multivariable analyses to adjust for potential baseline imbalances, such as the higher baseline RSBI observed in Group 4. Finally, our study utilizes an additive intervention model rather than a true factorial design. Because all active intervention groups received high-protein supplementation as a foundational nutritional therapy, the study design does not permit a clear separation of the independent contributions or interaction effects of high-protein supplementation, core muscle rehabilitation, and NMES. We cannot definitively determine the exact proportion of benefit derived from each specific modality. Although active interventions significantly improved weaning success, secondary outcomes (mortality and hospital length of stay) remained unchanged. PMV patients inherently suffer from severe comorbidities. While our targeted interventions successfully facilitated ventilator liberation, overall survival and discharge timelines in this fragile population are predominantly dictated by the trajectory of their primary underlying diseases. Nevertheless, this is the first study to investigate the clinical implications of different interventions in patients with PMV. Further larger, adequately powered multicenter randomized controlled trials with balanced allocation are required to confirm the independent and interactive efficacy of these interventions.

## Conclusions

In this pilot study, we demonstrated that high protein supplementation combined with core muscle rehabilitation and NMES programs are feasible non-invasive methods that may facilitate ventilator weaning in PMV patients. Lacking direct diaphragmatic measurements, the underlying mechanisms remain speculative. These preliminary findings require confirmation in adequately powered multicenter trials.

## Data Availability

The data will not be shared according to the regulations of Chang Gung Memorial Hospital IRB for patient confidentiality.
